# Prof. Dr. Erney Plessmann de Camargo (★1935 †2023)

**DOI:** 10.1590/0037-8682-0272-2023

**Published:** 2023-07-24

**Authors:** Luis Marcelo Aranha Camargo

**Affiliations:** 1 Universidade de São Paulo, Instituto de Ciências Biomédicas, Departamento de Parasitologia, São Paulo, SP, Brasil. Universidade de São Paulo Instituto de Ciências Biomédicas Departamento de Parasitologia São Paulo SP Brasil; 2 Secretaria de Estado da Saúde de Rondônia, Centro de Pesquisa em Medicina Tropical, Porto Velho, RO, Brasil. Secretaria de Estado da Saúde de Rondônia Centro de Pesquisa em Medicina Tropical Porto Velho RO Brasil; 3 Instituto Nacional de Ciência e Tecnologia de Epidemiologia da Amazônia Ocidental, Porto Velho, RO, Brasil. Instituto Nacional de Ciência e Tecnologia de Epidemiologia da Amazônia Ocidental Porto Velho RO Brasil

Erney Plessmann de Camargo, an only child, was born in Campinas, São Paulo, on April 20, 1935. He soon moved to the capital with his parents, who were simple people. They lived in Rua Barão de Limeira in the center of São Paulo. In addition to being a housewife, his mother, Dona Mary Marcondes Plessmann, managed a boarding house in her own home. His father, Felício Edgard de Camargo Cruz, was a career banker. A football lover, he adopted the São Paulo Futebol Clube as his favorite team. He attended elementary school in São Paulo, first at the João Coco Kopke School Group and later at the Liceu Coração de Jesus. At 17, he entered the Faculty of Medicine at USP.

In reality, he was much more concerned with Natural History than Medicine, perhaps influenced by a professor in the scientific course, Albretch Tabor (1901-1978). According to the professor, “When I entered the Faculty of Medicine in 1953, Medicine was still considered one of the centers of Natural History. It was still not common for people to study biology for science. Biology was more of a professor's career, and there was no such thing as a scientific career. Medicine offered the possibility of having a profession and eventually pursuing science in the natural sciences. That is more or less why I studied Medicine, I never had an irresistible medical vocation; what I liked was the aspect that I thought was the scientific component of medicine”.

**Figure:**
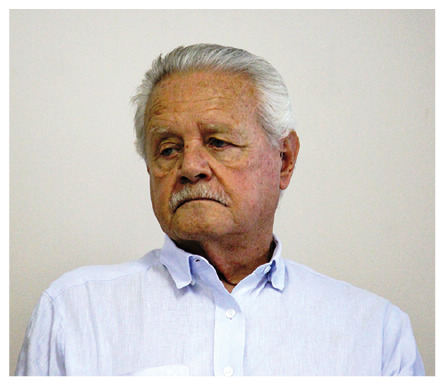

He then joined the Faculty of Medicine at USP as a professor. In 1964, with Institutional Act I of the Military Government, he was fired when working with the biochemical and molecular aspects of *Trypanosoma cruzi*^1^*,* having published his first independent work in 1963. He was married to Profa. Marisis Aranha Plessmann de Camargo who was a full professor, in her own right, at PUC/SP (the mainstay of career and that of her family). He was invited by Prof. Walter Plaut of the University of Wisconsin to fill an instructor position, the lowest level of the American college career. He then emigrated to the United States with his wife (with whom he lived for 63 years) and three children aged between two and six.

After five years, the then director of the Faculty of Medicine of Ribeirão Preto, Prof. José Moura Gonçalves, invited him and his research colleague Prof. Luiz Hildebrando Pereira da Silva, to set up a microorganism genetics department at USP in Ribeirão Preto.

In 1970 he moved to São Paulo with his wife and three children (and two years later, the youngest daughter would be born). To support himself, he worked at Editora Abril and the Lavoisier laboratory since his hiring by public institutions was prohibited due to the Military Government. After two years, Prof. Leal Prado invited him to work at the Escola Paulista de Medicina to develop research in the area of Microbiology and Parasitology, where he worked for 15 years, then joined as a full professor at the Institute of Biomedical Sciences (ICB) at USP in 1985 in the Department of Parasitology. Subsequently, under the management of Professor José Goldenberg, he assumed the Pro-Rectory of Research, a position to which he was reappointed twice more under the management of Prof. Roberto Lobo.

He then returned to the Parasitology department, making an effort to install an outpost in the Amazon (ICB5 / USP) in Rondônia^2^ in the early 1990s.

He was a manager at the Butantan Institute, INCOR, Oswaldo Cruz Institute, CAPES, AC Camargo Hospital, and CNPq. In 2005, he retired from the USP without ceasing to write scientific articles, guide students, or enthusiastically participate in activities in Rondônia. He was most recently the president of the Conrado Wessel Foundation, an institution concerned with promoting culture. During his teaching life, he supervised 15 master's students, eight doctoral students, and 11 postdoctoral students and participated in 22 co-supervisions and extensive scientific production.

During his journey, he received several Awards/Decorations: the Lafi Medicine Award, 1980; the Butantan Institute Medal, 2001; Samuel Pessoa Medal, from the Society of Protozoology, 2004; Commander, National Order of Scientific Merit, Brazil, 1996; Grand Cross, National Order of Scientific Merit, Brazil, 2002; Peacemaker Medal, from the Brazilian Army, 2003; Professor Emeritus, ICB-USP, 2005; Grand Cross of the Order of Ipiranga, 2006; Government of São Paulo, 2006; Doctor Honoris Causa, National University of Engineering, Lima, Pera, 2006; Tribute from the Brazilian Society of Tropical Medicine, 2009; Professor Emeritus, Faculty of Medicine, USP 2012; and Tribute ICB-USP, for work in the Amazon. 2019; Emeritus Professor at USP 2022.

The Professor left us at 2:38 pm on 3/3/2023, surrounded by family members, well cared for, and with as little suffering as possible. He is survived by his wife and four children, who are university professors and scientists.

Source: Circumstanced justification for the appointment of Prof. Erney Camargo as professor emetrite of USP written by Prof. Dr. Marco Antônio Zago and personal observations.
